# Clinical and genetic landscapes of mucopolysaccharidosis type III in 20 Chinese patients

**DOI:** 10.1186/s13023-026-04484-2

**Published:** 2026-07-31

**Authors:** Xueying Su, Yijing Zhang, Xiaoyuan Zhao, Xi Yin, Wen Zhang, Ye Song

**Affiliations:** 1 Department of Genetics and Endocrinology, Guangzhou Women and Children’s Medical Center, Guangzhou Medical University, Guangzhou, Guangdong China; 2 Department of Neurosurgery, Guangzhou Women and Children’s Medical Center, Guangzhou Medical University, Guangzhou, Guangdong China

**Keywords:** Mucopolysaccharidosis type III, Sanfilippo syndrome, Heparan N-sulfatase, α-N-acetylglucosaminidase, Glycosaminoglycan

## Abstract

**Background:**

Mucopolysaccharidosis type III (MPS III), also known as Sanfilippo syndrome, is a devastating lysosomal storage disorder characterized by severe, progressive neurodegeneration. Diagnosing this condition is challenging because early somatic signs are often mild, frequently leading to misdiagnosis as autism spectrum disorder or attention-deficit/hyperactivity disorder. As the subtype distribution and genetic mutation spectrum show significant ethnic heterogeneity, population-specific data are essential for accurate clinical management. This study aimed to investigate the clinical features, biochemical indicators, and genetic landscape of 20 Chinese patients (10 with type IIIA and 10 with type IIIB) to improve early recognition and diagnostic accuracy. Phenotypes and genotypes were summarized using urinary glycosaminoglycan levels, lysosomal enzyme activity assays, and whole-exome sequencing.

**Methods:**

A retrospective analysis was conducted on 20 patients with MPS III (10 with type IIIA and 10 with type IIIB). Urinary glycosaminoglycan (GAG) levels, lysosomal enzyme activities in peripheral blood leukocytes, and genetic analysis using whole-exome sequencing (WES) were performed to summarize their phenotypes and genotypes.

**Results:**

All 20 patients exhibited progressive neurodegeneration, with initial symptoms primarily including delayed language development (100%) and cognitive regression, accompanied by varying degrees of hyperactivity, sleep disorders, and coarse facial features. Biochemical data showed the majority of patients (18/20, 90%) had significantly elevated urinary GAG excretion (with two patients showing values within normal limits), with type IIIA patients showing markedly decreased activity of heparan N-sulfatase (SGSH) and type IIIB patients showing significantly reduced α-N-acetylglucosaminidase (NAGLU) activity. Genetic testing revealed that in the *SGSH* gene, c.1129C>T and c.703G>A were the most frequent mutations, while in the *NAGLU* gene, c.1693C>T and c.1081T>C were common mutations. Compared with European populations, the proportions of IIIA and IIIB subtypes were similar in this cohort.

**Conclusion:**

Chinese patients with MPS III exhibit significant clinical variability and delayed diagnosis. Delayed language development accompanied by mild facial dysmorphism is an important diagnostic clue. Population-specific high-frequency mutation sites exist in the *SGSH* and *NAGLU* genes, and clarifying the mutation spectrum can aid in precise genetic counseling and prenatal diagnosis.

**Supplementary Information:**

The online version contains supplementary material available at https://doi.org/10.1186/s13023-026-04484-2.

## Introduction

Mucopolysaccharidosis (MPS) is a group of autosomal recessive genetic disorders caused by deficiencies in specific lysosomal hydrolases, leading to the inability to properly degrade glycosaminoglycans (GAGs) and their progressive accumulation within tissue cells [1–4]. Based on the specific enzyme deficiency and clinical presentation, MPS is classified into 7 major types comprising 11 subtypes [5]. Compared to MPS types I, II, IV, VI, and VII, which exhibit significant multi-organ involvement (such as skeletal deformities and valvular disease), MPS III (also known as Sanfilippo syndrome) is characterized by severe and progressive central nervous system (CNS) degeneration [6].

In clinical practice, diagnosing MPS III poses a significant challenge. Early somatic signs in this type (such as coarse facial features and organomegaly) are relatively mild, and the initial symptoms are often language development delay, hyperactivity, and aggressive behavior, which are easily misdiagnosed as autism spectrum disorder or attention-deficit/hyperactivity disorder (ADHD), resulting in substantial diagnostic delays [7]. Regarding treatment, although enzyme replacement therapy (ERT) and hematopoietic stem cell transplantation (HSCT) have demonstrated efficacy for somatic symptoms in other MPS types, their benefit for the neurological symptoms of MPS III remains limited due to the blood-brain barrier [8–9]. Currently, novel approaches such as intrathecal ERT [10], substrate reduction therapy (e.g., miglustat) [11], and gene therapy [12–15] are under clinical investigation, aiming to overcome these therapeutic obstacles.

It is noteworthy that the subtype distribution and mutation spectrum of MPS III show significant ethnic and geographic heterogeneity [16]. While MPS III is often the most prevalent subtype in European populations, comprising 47% of cases in the Netherlands, 44% in Germany, and 48% in Poland, and it remains exceptionally rare in China [4, 16, 17]. The data from Chinese cohorts indicate that MPS II is the overwhelmingly dominant type (approximately 47.4%), whereas MPS III (subtypes IIIA and IIIB) has been reported to account for as little as 3.7% of the total MPS population. The children are more commonly misdiagnosed with idiopathic developmental delays, autism spectrum disorder, or ADHD. Consequently, many Chinese patients miss the critical window for early diagnosis and targeted multidisciplinary intervention, and the true burden of the disease remains underestimated. Therefore, a detailed analysis of the clinical phenotypes and genetic characteristics of Chinese MPS III patients is crucial for shortening the diagnostic timeline, optimizing clinical management, and enabling precise prenatal diagnosis. This study retrospectively analyzed the clinical data and gene mutation spectrum of 20 Chinese patients with MPS III, aiming to provide further evidence-based insights for the diagnosis and treatment of this disease in the Chinese population.

## Patients and methods

### Study design and ethical considerations

This study is a retrospective, single-center clinical analysis conducted at the Guangzhou Women and Children’s Medical Center. Ethical approval for this study (No. 2010-03) was obtained by the Institutional Review Board of Guangzhou Women and Children’s Medical Center. Informed consent was obtained from the parents or legal guardians of all participating patients in accordance with the tenets of the Declaration of Helsinki.

### Research objects

This study focused on 20 patients with MPS III (including 12 boys and 8 girls) who were admitted to the Genetics and Endocrinology Clinic of the Guangzhou Women and Children’s Medical Center between January 2014 and December 2024 for progressive neurological deterioration, coarse facial features, and multisystem involvement (e.g., hepatomegaly, hernia). All patients were from unrelated families (no siblings were included). 70% of the patients were from Guangdong Province, and the remaining 30% were from southern provinces surrounding Guangdong, including Guangxi, Hunan, Jiangxi, and Hainan.

### Clinical phenotypic and neurobehavioral assessment

Clinical evaluation encompassed assessments ranging from morphological characteristics to complex behavioral phenotypes. Rough face is defined as a typical appearance that combines characteristics such as tongue hypertrophy, thick eyebrows, protruding upper orbital margin, wide and flat nose bridge, forward nostrils, and thick lip valgus. In addition, developmental delay is determined by comparing whether the child has reached the milestone developmental indicators for the corresponding age group. For the purpose of this study, “age of onset” was defined as the age at which the first clinical symptoms attributable to MPS III were reported by caregivers or documented by physicians (most commonly language delay). “Age at diagnosis” was defined as the age at which the diagnosis was biochemically confirmed by specific enzyme activity assays followed by molecular confirmation at our medical center. All patients underwent a standardized clinical assessment at our center, including detailed medical history, physical examination, and neurobehavioral evaluation. However, not all patients received comprehensive systematic evaluations for every organ system (e.g., formal audiological testing, echocardiography, or skeletal surveys were performed based on clinical indications rather than as a universal protocol). The availability of specific investigations varied among patients, and prevalence proportions reported in the Results section are calculated based on the subset of patients for whom the relevant evaluation was performed.

### Biochemical metabolism and enzyme activity determination

Urinary glycosaminoglycan quantitative analysis was performed using the dimethylmethylene blue colorimetric assay [18]. Enzyme activities of heparan N-sulfatase and α-N-acetylglucosaminidase in peripheral blood leukocytes were determined using synthetic fluorescent substrates, following established protocols [19].

### Molecular genetic analysis and bioinformatics processes

Whole-exome sequencing was successfully performed on 17 patients. The remaining three patients (Cases 2, 3, and 11) could not be sequenced due to insufficient DNA quality or withdrawal of consent. Identified mutations were subsequently validated and parental origin was determined using Sanger sequencing (reference sequences: SGSH: NM_000199.5; NAGLU: NM_000263.4; HGSNAT: NM_152419.3; GNS: NM_002076.4).

### Statistical analysis

Data were analyzed using descriptive statistics. Continuous variables, such as age of onset and age at diagnosis, were expressed as medians or ranges. Categorical variables, including the gender distribution and prevalence of specific clinical symptoms, were expressed as frequencies and percentages. A difference with a p-value < 0.05 was considered as statistically significant difference. All statistical analyses were operated by Graphpad Prism 10.1.2.

## Result

### Basic information

**Fig. 1:**
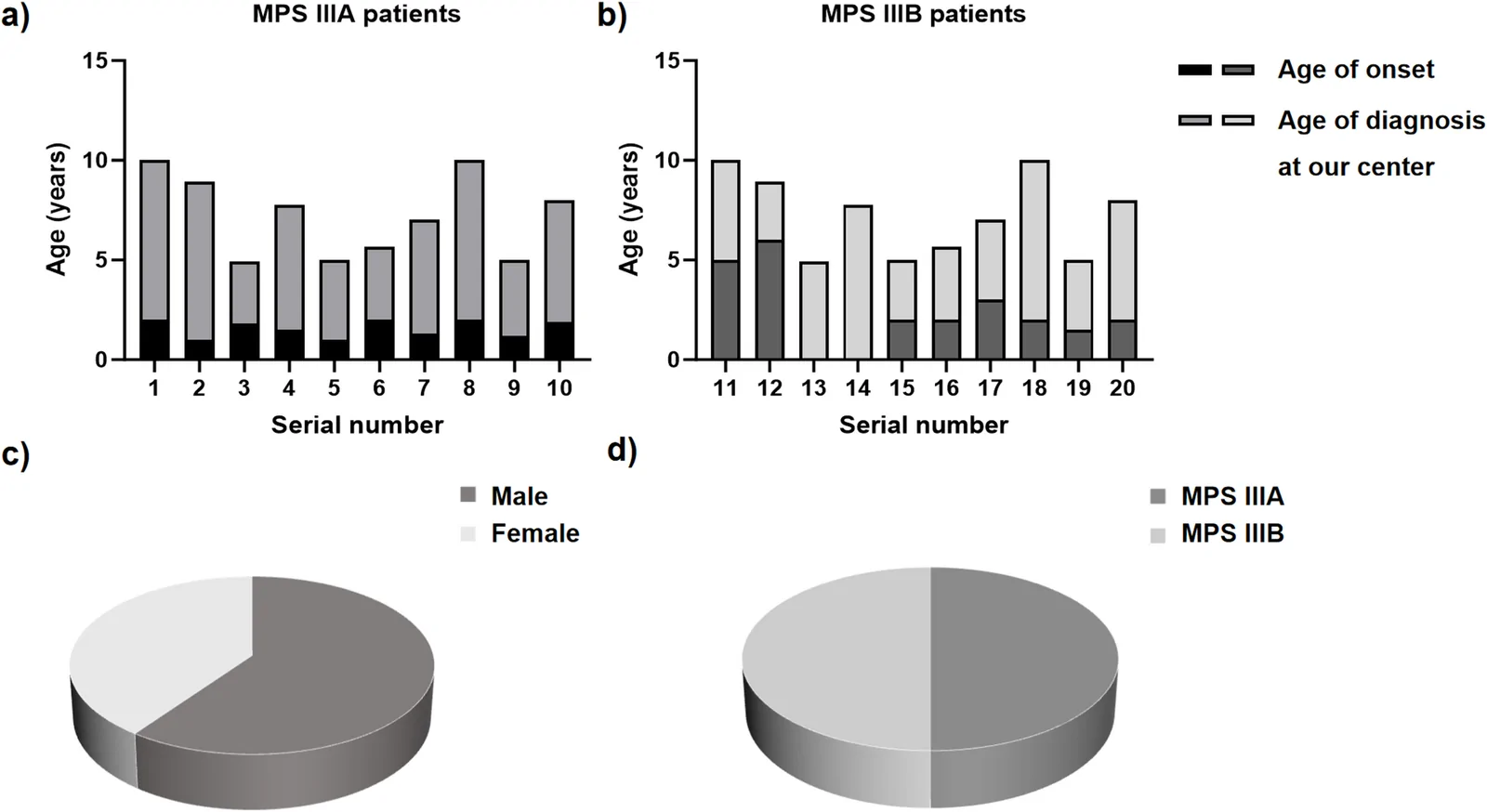
**a**-**b**) Age of onset and age at diagnosis for patients with MPS III. **c**-**d**) Gender and subtype distribution of MPS III patients. *The missing onset ages for Patients 13 and 14 are due to the limitations of retrospective data collection

Among 20 patients with MPS III, there were 12 males and 8 females (Fig. 1c). As shown in Fig. 1a and b, the onset age of patients with type IIIA was 1–2 years, and the age at diagnosis was 1–12 years. For type IIIB patients, the age of onset was 2–6 years, and the age at diagnosis was 5–10 years. Data on exact ages of onset were missing for Patients 13 and 14 due to incomplete retrospective records; these are indicated in Fig. 1b. The interval from symptom onset to definitive diagnosis was prolonged, indicating diagnostic difficulty and a high likelihood of misdiagnosis. This reflects that the current early screening system for the disease is not yet well-established, and early recognition of MPS IIIA and IIIB still faces considerable challenges, which may be related to atypical early symptoms and insufficient clinical awareness. Notably, MPS IIIA has an earlier onset (1–2 years) compared to type IIIB (2–6 years), reflecting differences in disease progression initiation between the two subtypes. A delayed diagnosis can deprive patients of the opportunity for early intervention, thereby adversely affecting their prognosis.

### Clinical manifestations

MPS III is primarily characterized by progressive neurological disorders, with symptoms gradually worsening with age; somatic features and visceral involvement are relatively mild. Based on the clinical presentation of this cohort, core symptoms such as neurological manifestations and distinctive facial features were universally present (Tables 1 and 2). Prevalence proportions for each clinical feature are reported based on the number of patients who underwent the relevant evaluation (denominator specified in each table). Not all patients completed every investigation. All patients exhibited stagnation or regression in language and intellectual development, which is the most central neurological feature of MPS III, indicating severe impairment of cognitive function. 70% of the patients (14/20) displayed characteristic mucopolysaccharidosis facial features, such as thick eyebrows, wide-set eyes, and a flattened nasal bridge, which serve as important clinical clues and are crucial for early suspicion. 45% of the patients (9/20) exhibited hyperactivity, aggressive behavior, or social deficits, suggesting a broad impact of the disease on behavioral development. Neuroimaging was not performed in all 20 patients because it was obtained based on clinical indications rather than as a universal screening protocol, as noted in the Methods section. Of the eight patients for whom neuroimaging data were available (3 with MPS IIIA and 5 with MPS IIIB), more than 85% (7/8) showed structural changes such as brain atrophy and ventricular enlargement, which underlie the neurological damage observed. Nearly one-third exhibited motor impairments, manifested as unsteady walking or regression in motor skills. The incidence of skeletal system abnormalities was 25% (5/20), with mild hepatosplenomegaly accounting for 20% (4/20). The incidence of adenoid hypertrophy and bilateral hernia were 16.7% (3/18) and 10% (2/20), respectively. Additionally, urinary incontinence was reported in two MPS IIIA patients. Cardiac involvement, when present, primarily affected the mitral and tricuspid valves.

**Table 1:** Statistics on the specific manifestations of progressive neurological damage in MPS Ⅲ

Clinical symptoms			Main Manifestations	MPS Ⅲ (*n* = 20)	MPS ⅢA (*n* = 10)	MPS ⅢB (*n* = 10)
Language/Intellectual Developmental Delay			Stagnation/regression of language and intellectual development, difficulty in communication, poor speaking ability compared with peers, and poor memory, etc.	100% (20/20)	100% (10/10)	100% (10/10)
Behavioral Abnormalities			Hyperactivity, inattention, destructive and aggressive behavior, social deficits, sleep disorders	45% (9/20)	40% (4/10)	50% (5/10)
Imaging Abnormalities			Brain atrophy, ventricular dilation, abnormal signals in cerebral white matter	87.5% (7/8)	100% (3/3)	80% (4/5)
Motor Disorders			Slow and unsteady walking, prone to falling, and motor regression	30% (6/20)	40% (4/10)	30% (3/10)
Other Abnormalities			Fecal and urinary incontinence occurring at 6 and 8 years old, respectively (2), Pineal cyst (1)	10% (2/20), 5% (1/20)	20% (2/10)	10% (1/10)

Percentages are calculated based on the number of patients with available data for each feature. Not all patients completed every investigation

**Table 2:** Physical characteristics and specific manifestations of visceral involvement in MPS III

Clinical symptoms	Main Manifestations	MPS Ⅲ (*n* = 20)	MPS ⅢA (*n* = 10)	MPS ⅢB (*n* = 10)
Special Facial Features	Coarse facial features, thick eyebrows, wide eye spacing, flat nasal bridge, thick nose, thick lips, enlarged head circumference	70% (14/20)	70% (7/10)	70% (7/10)
ENT Abnormalities	Hearing impairment (5), adenoid hypertrophy (3)	31.3% (5/16) 16.7% (3/18)	30% (3/10) 20% (2/10)	33.3% (2/6) 12.5% (1/8)
Skeletal System Abnormalities	Stiffness of elbow and finger joints, genu varum (O-shaped legs), multiple bone abnormalities, advanced bone age	25% (5/20)	40% (4/10)	10% (1/10)
Skin Abnormalities	Mongolian spots on the buttocks and back	25% (5/20)	40% (4/10)	10% (1/10)
Visceral Involvement	Mild hepatosplenomegaly	20% (4/20)	30% (3/10)	10% (1/10)
Gastrointestinal System Abnormalities	Bilateral inguinal indirect hernia, bilateral direct hernia	10% (2/20)	0 (0/10)	20% (2/10)
Cardiac Involvement	Sinus arrhythmia, frequent ventricular premature beats, mitral valve prolapse with regurgitation, abnormal echo of the anterior mitral leaflet, tricuspid regurgitation	8.3% (1/12)	0 (0/5)	20% (1/5)
Gonadal Developmental Abnormalities	Precocious puberty, abnormally enlarged testes	6.7% (1/15)	0 (0/5)	10% (1/10)

Percentages are calculated based on the number of patients with available data for each feature. Not all patients completed every investigation

**Fig. 2:**
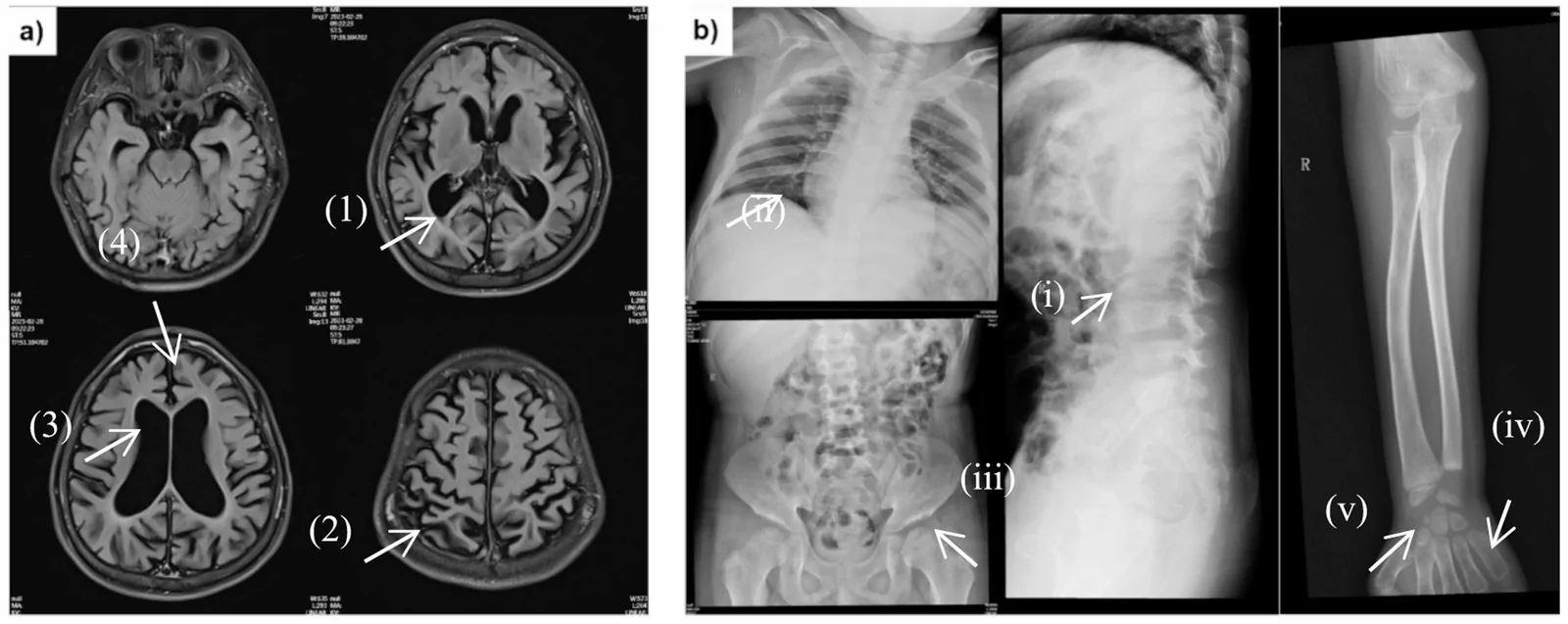
(**a**) Brain MRI and (**b**) skeletal radiographs from two patients with MPS IIIA. **a**) Brain MRI demonstrates findings of progressive diffuse cerebral atrophy. Arrows indicate: (1) marked enlargement of the lateral ventricles including temporal horns; (2) widened Sylvian fissures; (3) rounded anterior horns of lateral ventricles; (4) deepened and widened sulci at vertex levels giving a “walnut-like” appearance. **b**) Representative skeletal radiographs illustrate features of dysostosis multiplex. Arrows indicate: (i) vertebral ovoid shape and thoracolumbar anterior beaking; (ii) oar-shaped ribs (proximal narrowing with anterior spatulate widening); (iii) flared iliac wings and dysplastic acetabula; (iv) the broad and proximally pointed short metacarpals; (v) the V-shaped hypoplastic distal ulna and radius

Brain magnetic resonance imaging (MRI) and radiographic examinations of Patients 5 and 7, both with MPS IIIA, were shown in Fig. 2. Figure 2a (axial T2-FLAIR MRI) demonstrated diffuse brain atrophy, marked enlargement of the lateral ventricles (including temporal horns). As the imaging slices ascend, the Sylvian fissures were significantly widened, and the anterior horns of the lateral ventricles appear rounded and enlarged, reflecting progressive loss of frontal and temporal lobe parenchyma. At the level of the lateral ventricles, the images demonstrated severe ventriculomegaly, specifically an expansive “butterfly-like” enlargement, confirming compensatory ventricular enlargement (hydrocephalus ex vacuo) secondary to severe deep white matter reduction. At the vertex levels, diffuse cortical atrophy was evident, characterized by remarkably deepened and widened sulci alongside thin, shrunken gyri, manifesting a “walnut-like” appearance of the brain parenchyma. Collectively, these neuroimaging hallmarks signify the end-stage structural destruction caused by lysosomal storage-induced neuroinflammation and neuronal apoptosis in MPS III. The typical systemic skeletal involvement of dysostosis multiplex was displayed in Fig. 2b, which was a hallmark feature of mucopolysaccharidoses and other lysosomal storage disorders [20, 21]. Radiographic hallmarks included generalized vertebral ovalization and thoracolumbar anterior beaking, alongside the characteristic oar-shaped configuration of the ribs (proximal narrowing with anterior spatulate widening). Pelvic imaging identified flaring iliac wings, inferior iliac constriction, and dysplastic acetabula associated with coxa valga. Involvement of the extremities was marked by shortened, thickened long bones and distinctive hand anomalies: bullet-shaped metacarpals (proximally tapered) and short phalanges. Furthermore, the distal radius and ulna displayed an inward V-shaped obliquity, with articular surfaces tilting toward each other in a pattern typical of advanced lysosomal storage involvement.

### Biochemical indicators

The biochemical findings for the 10 MPS IIIA patients are presented in Table 3. The majority of patients (18/20, 90%) demonstrated elevated urinary GAG/creatinine ratios (GAG/Cr) above the age-adjusted reference limits; two patients (Patients 5 and 10) had values within the normal range. SGSH activity was profoundly deficient in every case, with measured values well below the normal range of 4.1–12.0 nmol/(mg·17 h). In the two patients with available comprehensive panels, biochemical profiles differed: Case 2 showed hypocholesterolemia alongside elevated transaminases (Alanine Aminotransferase/ Aspartate Aminotransferase, ALT/AST), suggestive of hepatic involvement, whereas Case 8 remained entirely unremarkable. In summary, the consistent biochemical profile of MPS IIIA in this cohort is defined by significantly increased urinary GAG excretion and a severe deficiency in SGSH enzyme activity.

**Table 3:** Biochemical indicators of MPS IIIA patients

Test Category	Case number Indicator	1	2	3	4	5	6	7	8	9	10	Reference Range
	Age	5y10m	10m16d	6y10m	4m19d	12y	5y9m	8y	5y10m	6y9m	3y7m	
Urinary Glycosamino-glycans	Urinary GAG (mg/L)	540.6	185.8	459.7	325.6	120.3	486.7	62.4	243	275.7	70.9	
	Urinary GAG/Cr (mg/mmol)	**65.7 (< 13.9)**	**98.3 (< 56.4)**	**33.9 (< 8.4)**	**64.4 (< 56.4)**	8.1 (< 8.4)	**30.8 (< 13.9)**	**28.5 (< 8.4)**	**50.0 (< 13.9)**	**22.5 (< 8.4)**	13.2 (< 13.9)	Reference range varies with age
Enzyme Activity	N-sulfoglucosamine sulfohydrolase Activity	**0.4**	**0.0**	**0.0**	**0.0**	**0.0**	**0.1**	**0.0**	**0.3**	**0.5**	**0.2**	4.1–12.0 nmol/(mg.17 h)
Biochemical Indicators	T-Chol (mmol/L)	/	**3.22**	/	/	/	/	/	4.05	/	/	3.4–5.2 mmol/L
	TG (mmol/L)	/	0.8	/	/	/	/	/	0.63	/	/	0.23–1.7 mmol/L
	ALT (U/L)	/	**52**	/	/	/	/	/	27	/	/	7–30 U/L
	AST (U/L)	/	**53**	/	/	/	/	/	32	/	/	14–44 U/L

Abnormal indicators were indicated in bold

As shown in Table 4, all patients with MPS IIIB exhibited significantly elevated urinary GAG/Cr levels compared to reference values (for example, patient 11 had a value of 39.7, far exceeding the reference value of < 8.4; patient 15 reached 60.4, far exceeding the reference value of < 13.9). Urinary GAG was also elevated to varying degrees, which is a typical biochemical manifestation of MPS, indicating severe disturbances in glycosaminoglycan metabolism in the body. The activity of α-N-acetylglucosaminidase was severely deficient, far below the reference range (5.0–22.0 nmol/(mg·17 h)). Abnormalities in multiple biochemical indicators reflect indirect involvement of various organs. For liver function, several patients showed elevated ALT and AST levels. As for blood lipids (Total Cholesterol (T-Chol) and Triglyceride (TG)), only a subset of patients showed abnormalities (such as patient 12 with elevated T-Chol, patient 19 with decreased T-Chol and elevated TG), suggesting that lipid metabolism is not primarily affected. Fasting blood glucose was slightly elevated only in patient 15, while most patients were normal, indicating no significant abnormalities in glucose metabolism.

**Table 4:** Biochemical indicators of MPS IIIB patients

Test Category	Case number Indicator	11	12	13	14	15	16	17	18	19	20	Reference Range
	Age	10y13d	8y11m	4y11m	7y9m	5y	5y8m	7y	10y	5y	8y	
Urinary Glycosamino-glycans	Urinary GAG (mg/L)	413.8	528.08	266.4	446.32	883	273.4	527.1	406.6	38.1	457.4	
	Urinary GAG/Cr (mg/mmol)	**39.7** **(< 8.4)**	**36.7** **(< 8.4)**	**38.2** **(< 13.9)**	**37.9** **(< 8.4)**	**60.4** **(< 13.9)**	**25.8** **(< 13.9)**	**24.4** **(< 8.4)**	**31.4** **(< 8.4)**	**29.4** **(< 13.9)**	**35.6** **(< 8.4)**	Reference range varies with age
Enzyme Activity	α-N-Acetylglucosaminidase Activity	**0**	**0**	**0**	**0**	**0**	**0**	**0**	**0**	**0**	**0**	5.0–22.0 nmol/(mg.17 h)
Biochemical Indicators	T-Chol (mmol/L)	/	**5.22**	/	/	4.63	/	4.05	5.14	**2.37**	3.73	3.4–5.2 mmol/L
	TG (mmol/L)	/	1.21	/	/	1.22	/	1.13	0.7	**2.31**	0.53	0.23–1.7 mmol/L
	ALT (U/L)	13	**37**	/	/	**45**	/	27	18	**41**	**33**	7–30 U/L
	AST (U/L)	27	29	/	/	39	/	24	20	27	32	14–44 U/L
	Fasting glucose (mmol/L)	5.68	4.9	/	/	**5.83**	/	5.54	5.57	5.11	5.23	3.5–5.7 mmol/L

Abnormal indicators were indicated in bold

### Molecular detection

Three patients did not undergo whole-exomesequencing due to insufficient sample quality or parental refusal; therefore, genetic data are available for the remaining 17 patients. The variants identified in these 17 patients are summarized in Table 5. A wide variety of mutation types were identified, including missense mutations (e.g., *SGSH*: p.Arg433Trp/Trp; *NAGLU*: p.Trp361Arg), nonsense mutations (e.g., *SGSH*: p.Glu140Ter; *NAGLU*: p.Arg626Ter), frameshift mutations (e.g., *SGSH*: p.H476Tfs*115), and splice-affecting variants (e.g., c.950–2A>G, c.679-1_679-5del5). Four novel variants were identified in MPS IIIA patients, indicated in bold in Table 5.

**Table 5:** Genetic characterization of MPS III patients

Case number	MPS III subtype	Gene	HGVS (DNA variant)	HGVS (protein variant)	ACMG classification
1	IIIA	*SGSH*	**c.570C>G** / **c.642del**	p.(Tyr190Ter) / p.(Tyr215ThrfsTer49)	Likely Pathogenic / Pathogenic
4	IIIA	*SGSH*	**c.570C>G** / c.1483T>C	p.(Tyr190Ter) / p.(Cys495Arg)	Likely Pathogenic / Likely Pathogenic
5	IIIA	*SGSH*	c.1129C>T / c.1411T >C	p.(Arg377Cys) / p.(Trp471Arg)	Pathogenic / Likely Pathogenic
6	IIIA	*SGSH*	**c.458C>T** / c.1298G>A	p.(Thr153Ile) / p.(Arg433Gln)	Likely Pathogenic/ Pathogenic
7	IIIA	*SGSH*	**c.1426del** / c.1418G>A	p.(His476ThrfsTer115) / p.(Trp473Ter)	Pathogenic / Pathogenic
8	IIIA	*SGSH*	**c.418G>T** / **c.950–2A>G**	p.(Glu140Ter) / (splice site)	Pathogenic / Likely Pathogenic
9	IIIA	*SGSH*	c.1297C>T / c.1063G>A	p.(Arg433Trp) / p.(Glu355Lys)	Pathogenic / Pathogenic
10	IIIA	*SGSH*	c.1297C>T / **c.570C>G**	p.(Arg433Trp) / p.(Tyr190Ter)	Pathogenic / Likely Pathogenic
12	IIIB	*NAGLU*	c.926A>G / c.1693C>T	p.(Tyr309Cys) / p.(Arg565Trp)	Pathogenic / Pathogenic
13	IIIB	*NAGLU*	c.309G>A / c.1081T>C	p.(Trp103Ter) / p.(Trp361Arg)	Pathogenic / Pathogenic
14	IIIB	*NAGLU*	**c.679-5_679-1del5** / c.1354G>A	(splice site) / p.(Glu452Lys)	Pathogenic / Pathogenic
15	IIIB	*NAGLU*	c.1693C>T / c.1910_1912delTCT	p.(Arg565Trp) / p.(Phe637del)	Pathogenic / Pathogenic
16	IIIB	*NAGLU*	c.1876C>T / c.2020C>T	p.(Arg626Ter) / p.(Arg674Cys)	Pathogenic / Pathogenic
17	IIIB	*NAGLU*	c.1081T>C / c.1946G>C	p.(Trp361Arg) / p.(Trp649Ser)	Pathogenic / Likely Pathogenic
18	IIIB	*NAGLU*	c.1562C>T / c.2020C>T	p.(Pro521Leu) / p.(Arg674Cys)	Pathogenic / Pathogenic
19	IIIB	*NAGLU*	c.1562C>T / c.1562C>T	p.(Pro521Leu) / p.(Pro521Leu)	Pathogenic / Pathogenic
20	IIIB	*NAGLU*	c.700C>T / c.1562C>T	p.(Arg234Cys) / p.(Pro521Leu)	Pathogenic / Pathogenic

Genetic data were obtained for 17 of 20 patients (Cases 2, 3, and 11 did not undergo whole-exome sequencing due to insufficient sample quality or parental refusal). Novel variants are indicated in bold. ACMG classification follows the 2015 ACMG/AMP guidelines

To provide a broader epidemiological context, Supplementary Tables S1 and S2 summarize the comprehensive literature review of all previously reported *SGSH* and *NAGLU* mutations in the Chinese population. In the *SGSH* gene, a total of 17 different mutations were observed, among which c.1129C>T and c.703G>A each appeared 5 times, making them the most frequent mutations; followed by c.1201G>C, c.571G>A, and c.544C>T, each observed twice; the remaining mutations were detected only once, including one heterozygous deletion of Exons 1–8. In the mutation spectrum of the *NAGLU* gene (corresponding to MPS IIIB), c.1693C>T appeared 5 times with the highest frequency; c.1081T>C and c.1004A>G were each observed 4 times; several mutations (such as c.26C>A, c.1876C>T, c.999_1019dup, etc.) appeared twice; most other mutations were found only once, indicating that mutations in this gene are relatively dispersed.

### Genotype-phenotype correlations

In our cohort, two recurrent mutations allowed within-gene comparisons of genotype-phenotype correlations. For *NAGLU* p.Pro521Leu (c.1562C>T), three unrelated patients exhibited different clinical courses depending on zygosity and the second allele. The homozygous patient (Case 19) had the earliest onset (2 years), youngest diagnosis (5 years), and most rapid motor decline (wheelchair by 7 years), consistent with previous reports that homozygosity for this mutation causes a severe phenotype [22, 23]. In contrast, compound heterozygotes with p.Arg674Cys (Case 18) or p.Arg234Cys (Case 20) presented later (3–4 years) and showed slower progression, yet all three had similarly elevated urinary GAG/Cr levels (29–36 mg/mmol), indicating that GAG excretion alone does not predict disease severity. For *SGSH* p.Arg433Trp (c.1297C>T), two patients shared the same mutation but differed in their second allele. Case 9 (second allele p.Glu355Lys) and Case 10 (second allele p.Tyr190Ter) both had early onset (1-1.5 years) and profoundly reduced SGSH activity (< 5% of normal). However, Case 10, carrying a nonsense second allele, had lower residual enzyme activity (0.2 vs. 0.5 nmol/(mg.17 h)), and additionally presented with hearing impairment and hepatomegaly, which were absent in Case 9. These observations, although limited by small sample size, highlight that the second allele and zygosity substantially modify the phenotype.

## Discussion

Our study shows an equal distribution of MPS IIIA and IIIB (*n* = 10 each) in this Chinese cohort, consistent with previous reports from mainland China [24]. This balanced ratio contrasts with the predominance of type IIIA in European populations and the exceptionally high frequency of type IIIB (86%) reported in Taiwan [17]. These differences demonstrate clear geographic and ethnic clustering of mutations - specifically, the prevalence of *SGSH* variants in Northern Europe versus the higher frequency of *NAGLU* mutations in Greece and regions of Asia. Given the estimated global prevalence of 1–9 per million, China’s vast population likely harbors a substantial undiagnosed burden. Establishing a comprehensive national registry through expanded screening and longitudinal tracking remains essential to define the true epidemiology of MPS III in China.

Neurocognitive and linguistic regression were ubiquitous in our cohort, with half exhibiting concurrent hyperactive or aggressive behaviors. This clinical profile, where profound central nervous system deterioration overshadows the subtle somatic signs common in types I and II, such as coarse facies and skeletal dysplasia, typifies the diagnostic challenge of MPS III. Indeed, the protracted interval between symptom onset and definitive diagnosis in this series highlights a pervasive “diagnostic odyssey.” Early behavioral disturbances are frequently misdiagnosed as primary autism or idiopathic ADHD, delaying appropriate intervention. Our findings advocate for an immediate metabolic workup in any child presenting with the dual burden of developmental regression and hyperkinetic behavior. Previous reports by Kong et al. (2020) noted hepatosplenomegaly and dental serration as potential hallmarks for MPS IIIB in Chinese patients [24]. In our cohort, we also incidentally observed elevated ALT and mild hepatic involvement in a subset of patients. However, given our relatively small sample size and the retrospective nature of the biochemical workup, whether these hepatic markers can reliably serve as early diagnostic clues for subtype differentiation remains inconclusive and requires validation in larger, systematic studies. Ultimately, pediatricians must maintain a low threshold for suspicion when encountering the “coarse facies-regression” dyad to mitigate current diagnostic shortfalls.

In our cohort, the *SGSH* variants c.1129C>T and c.703G>A were most frequent in MPS IIIA, consistent with the mutational spectrum previously identified in Chinese populations. The c.703G>A variant is notably associated with a severe phenotype when homozygous, which correlates with severe phenotypes in the homozygous state. For MPS IIIB, the *NAGLU* variant c.1693C>T (p.R565W) was predominant - likely attributable to its location within a highly mutable CpG hotspot [25]. However, while CpG hypermutability can generate identical variants independently in unrelated populations [26], a founder effect cannot be excluded for this mutation in specific geographical regions without formal haplotype analysis.

Distinguishing between mutational hotspots and founder effects is critical for interpreting recurrent mutations in a given population [27]. Mutational hotspots arise from intrinsically high mutation rates (e.g., CpG deamination), whereas founder effects result from population bottlenecks or genetic drift that propagate a rare allele within a specific ethnic subgroup [27]. In our Chinese cohort, some recurrent mutations (e.g., *SGSH* c.1297C>T and *NAGLU* c.1693C>T) are likely true mutational hotspots due to CpG dinucleotide hypermutability [22, 27]. Conversely, others (e.g., *SGSH* c.703G>A and NAGLU c.1081T>C) may reflect founder effects given their restricted geographic and population distribution in Chinese subpopulations [24]. Notably, the *NAGLU* c.700C>T (p.Arg234Cys) variant provides a compelling example of how the same mutation can represent a founder effect in one population (Iberian Peninsula) while possibly arising from distinct events in others [28], underscoring the need for population-specific analysis. We fully acknowledge that this question cannot be definitively resolved with our current data and should be addressed by future studies combining haplotype analysis with functional assays.

In the absence of approved disease-modifying therapies, management of MPS III currently focuses on supportive and symptomatic care. Early diagnosis enables genetic counseling, family planning, timely referral for supportive interventions, and identification of eligible patients for emerging clinical trials, rather than implying neurocognitive preservation or reversal [29]. While not curative, timely initiation of these supportive measures is a critical early intervention that significantly maximizes the patient’s quality of life. In China, HSCT is available at major tertiary referral centers, and since 2024, the National Healthcare Security Administration has progressively expanded reimbursement for HSCT for specific hematologic indications. However, HSCT is not a reimbursed indication for MPS III given the lack of evidence for neurocognitive benefit, consistent with international consensus guidelines [29]. Hence, the field has shifted toward more precise platforms, including CRISPR/Cas9, AAV-mediated delivery, and mRNA-LNP systems. The clinical viability of such strategies is underscored by the progress of RGX-121 (AAV9.CB7.hIDS). By targeting the CNS via a single intrathecal dose, this MPS II candidate has successfully met primary endpoints in pivotal trials [30]. For Sanfilippo syndrome, these advancements provide a translational roadmap for achieving sustained, localized transgene expression, moving the field closer to a definitive genetic solution.

The limitations of this study are as follows. This study is a single-center retrospective case series, which may limit the generalizability of the findings to the entire Chinese population given the geographic size and population heterogeneity of China. The patients were predominantly from southern China (Guangdong and neighboring provinces), and regional differences in diagnostic practices and access to specialist care could influence the reported subtype distribution and mutation frequencies. Furthermore, not all patients underwent complete systematic evaluations for all organ systems, which may have led to underascertainment of certain clinical features. The small sample size precludes robust statistical analyses of genotype-phenotype correlations. Future multi-center prospective studies with standardized protocols and national patient registries are needed to validate and extend our observations.

## Conclusion

This study of 20 Chinese patients delineates a distinct clinical and genetic landscape for MPS III, characterized by an equivalent frequency of subtypes IIIA and IIIB. The characteristic neurocognitive regression, frequently masked by mild somatic features, perpetuates a prolonged diagnostic odyssey. Identification of high-frequency mutation hotspots in *SGSH* and *NAGLU* offers a framework for efficient molecular screening and precise genetic counseling in the Chinese population. These findings justify a high index of suspicion and early metabolic evaluation for patients presenting with both developmental delay and hyperkinesia. Looking ahead, establishing a national patient registry and advancing next-generation gene therapy platforms remain critical to bridging the existing therapeutic gap and improving long-term clinical outcomes in China.

## Supplementary Information

Below is the link to the electronic supplementary material.

Supplementary Material 1

## Data Availability

Data from patients can be made available from the corresponding author after discussion with the Institutional Review Board. Consent from participants for data sharing was not obtained but any shared data will be anonymised.

## Abbreviations

MPS: Mucopolysaccharidosis
GAGs: Glycosaminoglycans
CNS: Central nervous system
ADHD: Attention-deficit/hyperactivity disorder
ERT: Enzyme replacement therapy
HSCT: Hematopoietic stem cell transplantation
WES: Whole-exome sequencing
MRI: Magnetic resonance imaging
ALT: Alanine Aminotransferase
AST: Aspartate Aminotransferase
T-Chol: Total Cholesterol
TG: Triglyceride
