# Supplementary material for: Clinical and genetic landscapes of mucopolysaccharidosis type III in 20 Chinese patients
Source: Orphanet J Rare Dis. 2026 Jul 31;21:386. doi: 10.1186/s13023-026-04484-2 (PMC13733165; doi:10.1186/s13023-026-04484-2)
Supplement: Supplementary file 1 [file 13023_2026_4484_MOESM1_ESM.docx]

Table S1. Frequency of *SGSH* mutations in Chinese patients reported with MPS IIIA based on literature review.

| **Gene** | Mutations of gene (c.DNA) | Frequency | Literature sources |
| --- | --- | --- | --- |
| *SGSH* | c.1129C>T | 5 | [1, 2, 3] |
| *SGSH* | c.703G>A | 5 | [1, 2, 3, 4] |
| *SGSH* | c.1201G>C | 2 | [1] |
| *SGSH* | c.571G>A | 2 |  |
| *SGSH* | c.544C>T | 2 |  |
| *SGSH* | c.302T>C | 1 |  |
| *SGSH* | c.1298C>T | 1 |  |
| *SGSH* | c.416C>T | 1 |  |
| *SGSH* | c.448C>T | 1 |  |
| *SGSH* | c.1225delC | 1 |  |
| *SGSH* | c.126C>A | 1 | [2] |
| *SGSH* | c.877C>T | 1 |  |
| *SGSH* | c.863C>T | 1 | [3] |
| *SGSH* | c.1063G>T | 1 |  |
| *SGSH* | c.1235T>C | 1 |  |
| *SGSH* | c.97G>A | 1 |  |
| *SGSH* | Exon 1–8 heterozygous deletion | 1 |  |

Table S2. Frequency of *NAGLU* mutations in Chinese patients reported with MPS IIIB based on literature review.

| **Gene** | Mutations of gene (c.DNA) | Frequency | Literature sources |
| --- | --- | --- | --- |
| *NAGLU* | c.1693C>T | 8 | [1, 2, 5] |
| *NAGLU* | c.1081T>C | 5 | [1, 2, 5] |
| *NAGLU* | c.1004A>G | 4 | [1, 2] |
| NAGLU | c.1694G>A | 3 | [5] |
| *NAGLU* | c.26C>A | 2 | [1, 2] |
| *NAGLU* | c.1876C>T | 2 |  |
| *NAGLU* | c.999_1019dupCGTCTATGAGGCCATGACTGC | 2 |  |
| *NAGLU* | c.700C>T | 2 | [2, 5] |
| *NAGLU* | c.383+1G>T | 2 | [2] |
| *NAGLU* | c.388C>T | 2 |  |
| *NAGLU* | c.2020C>T | 1 | [1] |
| *NAGLU* | c.1041G>A | 1 |  |
| *NAGLU* | c.2171_2172delTG | 1 |  |
| *NAGLU* | c.402T>G | 1 |  |
| *NAGLU* | c.993_994insACTGCCGTCTATGAGGCCATG | 1 |  |
| *NAGLU* | c.309G>A | 1 |  |
| *NAGLU* | c.2010C>G | 1 |  |
| *NAGLU* | c.1910_1912del | 1 |  |
| *NAGLU* | c.407G>C | 1 |  |
| *NAGLU* | c.1946G>C | 1 |  |
| *NAGLU* | c.587C>T | 1 |  |
| *NAGLU* | c.1676A>T | 1 |  |
| *NAGLU* | c.399_414del16 | 1 |  |
| *NAGLU* | 679-5_679-1delTCCAG | 1 |  |
| *NAGLU* | c.252_253ins19 | 1 | [2] |
| *NAGLU* | c.1493T>C | 1 |  |
| *NAGLU* | c.926A>G | 1 |  |
| *NAGLU* | c.1241A>G | 1 |  |
| *NAGLU* | c.660delC | 1 |  |
| *NAGLU* | c.764+1G>A | 1 |  |
| *NAGLU* | c.994_1014dup | 1 |  |
| *NAGLU* | c.461T>G | 1 |  |
| NAGLU | c.874G>A | 1 | [5] |
| NAGLU | c.867delC | 1 |  |

Note: Unlike Table 5 in the main text, which exclusively details the genotypes of the 20-patient cohort identified in the current study, these supplementary tables compile all variants previously reported in the broader Chinese population context based on a comprehensive literature review.

Reference

[1] Kong W, Meng Y, Zou L, Yang G, Wang J, Shi X. Mucopolysaccharidosis III in Mainland China: natural history, clinical and molecular characteristics of 34 patients. J Pediatr Endocrinol Metab. 2020; 33(6):793-802.

[2] Lin H, Chuang C, Lee C, Tu R, Lo Y, Chiu P, Niu D, Fang Y, Chen T, Tsai F, Hwu W, Lin S, Chang T, Lin S. Mucopolysaccharidosis III in Taiwan: Natural history, clinical and molecular characteristics of 28 patients diagnosed during a 21-year period. Am J Med Genet A. 2018; 176(9):1799-1809.

[3] Liu J, Liu F, Qiao G. Gene mutation spectrum and effects of hematopoietic stem cell transplantation in patients with mucopolysaccharidosis type III. Modern Journal of Integrated Traditional Chinese and Western Medicine. 2023,32(19):2712-2716 (in Chinese).

[4] Pan L, Su W, Lin D. Diagnosis and prenatal diagnosis of a rare mucopolysaccharidosis type Ⅲ family. Hainan Medical Journal, 2020, 31(15):4 (in Chinese).

[5] Tang J, Pan J, Guo Y, Ai Y, Jiang W, Du M, Fang Q. Mucopolysaccharidosis type ⅢB mutations in Chinese patients: identification of two novel NAGLU mutations and analysis of two cases involving prenatal diagnosis. Clin Chim Acta. 2013; 419:33-8.
